# Elevated plasma biomarkers of inflammation in acute ischemic stroke patients with underlying dementia

**DOI:** 10.1186/s12883-020-01859-1

**Published:** 2020-08-05

**Authors:** Dennis W. Choi, Tae Song Kim, Young Soo Kim, Dong Jin Kim

**Affiliations:** 1 Department of Neurology, Health Sciences Center T12-020, SUNY Stony Brook, Stony Brook, NY 11794 USA; 2 Brain Science Institute, Korea Institute of Science and Technology, 5, Hwarang-ro 14-gil, Seongbuk-gu, Seoul, 02792 South Korea; 3 Department of Pharmacy, Yonsei University, 85 Songdogwahak-ro, Yeonsu-gu, Veritas Hall D411, Incheon, 21983 South Korea

**Keywords:** Biomarkers, Blood-brain barrier, Inflammation, Dementia, Alzheimer’s disease, Diagnosis, Blood-test, Interleukin-6, C-reactive protein, S100B protein, Complement C3

## Abstract

**Background:**

The blood-brain barrier has been a hindrance to developing blood-based diagnostic tests for dementias, as it limits the appearance of brain biomarkers in the blood. Our aim was to see if the natural opening of the blood-brain barrier induced by ischemic stroke would increase serum levels of inflammatory biomarkers known to be elevated in the brains of patients with Alzheimer’s disease and other neurodegenerative dementias.

**Methods:**

Forty-three patients with acute ischemic stroke presenting to Stony Brook University Hospital were prospectively enrolled in the study. Eight of these patients were clinically diagnosed as having an underlying neurodegenerative dementia. Blood was drawn acutely within 72 h of stroke symptom onset, and serum levels of the classic inflammatory biomarkers, interleukin-6 (IL-6) and C-reactive protein (CRP) were measured, along with levels of S100B protein (S100B) and complement C3 (CC3).

**Results:**

Serum levels of IL-6 and CRP in patients with acute ischemic stroke and underlying dementia (AIS + D) were significantly higher (*p* = 0.002 and 0.003, respectively) than in patients with acute ischemic stroke alone (AIS). Serum levels of S100B and CC3 did not differ significantly between the groups.

**Conclusions:**

This study supports the possibility that opening of the blood-brain barrier may enhance the blood appearance of brain tissue markers of inflammation associated with neurodegenerative dementia. Further study is warranted to test this possibility, given the recent emergence of methods to open the blood-brain barrier for diagnostic or therapeutic purposes.

## Background

Diagnostic tests capable of identifying patients with Alzheimer’s disease (AD) and other neurodegenerative dementias are much needed. The ideal diagnostic test would accurately reveal disease attributes across all tested populations, while being convenient, inexpensive, and safe. Such an ideal test for AD still lies in the future, but there has been progress in developing CSF-based biochemical tests [1] and positron emission tomography (PET) tests utilizing ligands for amyloid β protein or tau protein [2]. Blood-based tests would be preferable to these in terms of cost and patient acceptance, but have been challenging to develop, other than genetic tests for familial disorders. A systematic hinderance has been the blood-brain barrier (BBB), which limits the appearance of brain tissue markers in the blood. For example, the putative Alzheimer’s disease marker p-tau181 (tau protein phosphorylated at threonine 181), is present in CSF at higher concentrations than in plasma [3].

Recently a practical and non-invasive method for transiently opening the mammalian BBB has been identified: low intensity focused ultrasound (FUS). The targeted application of ultrasonic pressure waves, at intensities lower than those used to ablate tissue, can reversibly disrupt brain capillary endothelial cell tight junctions for hours, permitting therapeutic drugs or nanoparticles to pass from the circulation to a desired region of brain tissue [4, 5]. The safety of low intensity FUS has been supported by animal studies, and clinical trials are underway [6]. While low intensity FUS has primarily been considered in therapeutic contexts, its ability to open the BBB might increase brain tissue biomarker levels in plasma and thus aid the blood-based diagnosis of brain disorders.

We performed a clinical study to seek preliminary evidence supporting this line of thought, specifically asking whether the BBB breakdown invariably associated with acute ischemic stroke [7] was associated with increased plasma levels of the classic inflammatory biomarkers interleukin-6 (IL-6) and C-reactive protein (CRP) in patients carrying a clinical diagnosis of dementia. IL-6 associates with its receptor, IL-6R and glycoprotein 130 to activate both classic and trans-signaling, enhancing inflammation and contributing to the pathogenesis of multiple diseases of the body and brain [8]. IL-6 and other proinflammatory cytokines elevate the expression and secretion of the pentraxin acute-phase protein, CRP, a component of the soluble innate immune system [9].

Chronic neuroinflammation is a core feature of many neurodegenerative diseases [10], including specifically AD [11]. Central nervous system IL-6 is upregulated in AD brain tissue [12] and has been implicated in modifying the pathogenesis of AD, Parkinson’s disease, and Huntington’s disease [13, 14]. Similarly, CRP mRNA expression is upregulated in AD brain to an extent rivaling that of liver expression [15], and CRP protein is associated with AD plaques and tangles [16]. However, despite broad evidence for upregulation of these brain inflammation markers in AD and other neurodegenerative dementias, a recent large meta-analysis of available studies concluded that ordinary peripheral blood levels of IL-6 and CRP in elderly patients with AD did not differ from that of controls [17].

## Methods

Patients presenting with acute ischemic stroke to the Comprehensive Stroke Center at Stony Brook University Hospital (Stony Brook, NY) between July, 2014 and March, 2015 were prospectively enrolled in this study. Immediate admission evaluations were performed by university stroke service physicians with board-certification in neurology and fellowship training in vascular neurology. Inclusion criteria were: 1) stroke service diagnosis of acute stroke with onset within the past 72 h; and 2) MRI confirmation of acute ischemic stroke. If a study coordinator was available, the coordinator obtained informed consent from the patient (or a legally authorized representative) and oversaw the collection of a study blood sample, usually during a venipuncture necessary for other initial or early clinical blood tests.

The blood sample was collected in a red-top (no additive) glass tube, and serum was conventionally extracted by the hospital clinical laboratory, frozen, assigned a code number, and sent to a commercial clinical laboratory (ARUP Laboratories, Salt Lake City, Utah, United States) for measurement of IL-6 and CRP (high sensitivity), as well as for complement component 3 (CC3) and S-100B protein (S-100B). The lower limit of detection for the IL = 6 assay was 5 pg/mL, with a normal value < 5 pg/mL. ARUP’s normal value for CRP was 3 mg/L or less; for S-100B protein, 0–96 ng/L; for CC3, 88–201 mg/dL.

The study coordinator then reviewed the medical records of each enrolled patient to determine whether the stroke service had additionally diagnosed the patient with a progressive neurodegenerative dementia (including specifically Alzheimer’s disease) during the patient’s hospital stay. In most cases, these diagnoses had been previously made by patients’ regular outside physicians on clinical criteria. If the stroke service physicians concurred, these diagnoses were added to the inpatient medical record. Coordinator clinical review took place prior to obtaining blood testing results.

A target enrollment of 50 patients with acute ischemic stroke was prospectively sought. Assuming 10–15% prevalence of dementia of in these patients (Pendlebury 2019; Plassman 2007), this design was considered likely to yield around 5–8 patients with acute ischemic stroke occurring on top of a pre-existing neurodegenerative dementia (AIS + D), and about 40 control patients with acute ischemic stroke but no pre-existing dementia (AIS). Retrospectively, a decision was made to exclude patients that were diagnosed with a concurrent active infection, autoimmune disease or active cancer, as these disorders are independently associated with elevated serum IL-6 and/or CRP. As a result of this additional criterion, 4 enrolled patients were excluded from further analysis: one with fever and leukocytosis; one with a recent diagnosis of temporal arteritis; and two with active lung cancer. Three more enrolled patients did not complete laboratory testing due to errors (serum samples inadequate or lost).

For all patients, age, sex, and past medical history was tabulated, included past history of atrial arrhythmia, chronic kidney disease, hyperlipidemia, hypertension, congestive heart failure, diabetes, coronary heart disease, obesity, hypothyroidism, smoking or established chronic obstructive pulmonary disease, and Parkinson’s disease. Initial National Institute of Health stroke scale (NIHSS) scores at presentation, and time elapsed from symptom onset to study venipuncture, were also determined.

After breaking study blind, statistical analysis of clinical and laboratory data was performed using R version 3.6.1 (R Foundation for Statistical Computing, Vienna, Austria). In Table 1, parametric values were presented as mean ± standard deviation (SD), and non-parametric values were presented as median and range between 25th percentile and 75th percentile values. For continuous variables, comparison between AIS + D and AIS groups was performed using the two-tailed Student *t* test or the Mann-Whitney *U* test as appropriate, after testing for normality with the Shapiro-Wilk normality test. Comparisons between categorical variables were performed using the *Χ*^2^ test. A *p* value < 0.05 was considered significant.

**Table 1:** Clinical and laboratory data by group

	AIS (*n* = 35)	AIS + D (*n* = 8)	χ^2^	*p*
Age	64.5 ± 14.1	85.8 ± 9.6		0.0003
Sex			0.73	0.39
Male	27 (77%)	5 (63%)		
Female	8 (23%)	3 (37%)		
Time from stroke onset to venipuncture (h)	44.4 ± 17.5	39.5 ± 20.9		0.53
Initial NIHSS	4.3 ± 4.3	11.9 ± 8.1		0.009
Other conditions				
Atrial arrhythmia	7 (20%)	6 (75%)	9.34	0.002
Kidney disease	4 (11%)	0 (0%)	1.01	0.32
Hyperlipidemia	21 (60%)	4 (50%)	0.27	0.61
Heart failure	3 (9%)	4 (50%)	8.20	0.004
Diabetes	7 (20%)	1 (3%)	0.24	0.62
Coronary artery disease	5 (14%)	1 (13%)	0.02	0.90
Obesity	2 (6%)	0 (0%)	0.48	0.49
Hypothyroidism	3 (9%)	0 (0%)	0.74	0.39
Smoking / COPD	6 (17%)	2 (25%)	0.27	0.61
Gastritis / ulcer	4 (11%)	0 (0%)	1.01	0.32
Parkinson’s disease	0 (0%)	1 (13%)	4.48	0.03
IL-6 (pg/mL)	0 (0–5.5)	11.5 (9.8–18.0)		0.002
CRP (mg/L)	3.5 (1.6–9.6)	19.8 (13.2–41.8)		0.003
S100B (ng/L)	55.0 (46.0–91.5)	94.5 (71.8–464)		0.065
CC3 (mg/dL)	130 (111–142)	111 (105–123)		0.16

Note: Age is presented as mean ± standard deviation. Laboratory values are presented as median and range between 25th percentile and 75th percentile values, with between group comparisons performed using the Mann-Whitney *U* test

## Results

A total of 43 acute ischemic stroke patients were enrolled and analyzed. Eight were clinically diagnosed by the stroke service as having an underlying progressive neurodegenerative dementia (AIS + D) and 35 had no such diagnosis of neurodegenerative dementia (AIS). Demographic and clinical features of these patients, as well as the results of blood tests, are presented in Table 1.

**Fig. 1:**
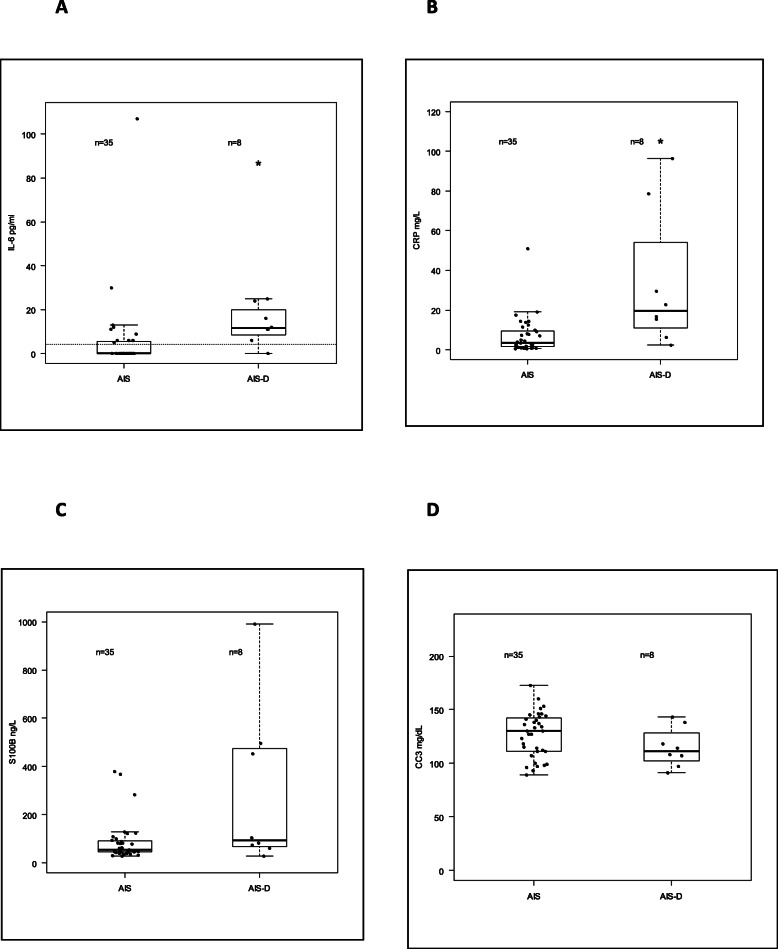
Combined dot and box plots of laboratory data in AIS (ctrl) and AIS + D (d) groups. Undetectable levels of IL-6 (< 5 pg/mL) were assigned a value of 0 but a dotted line at is drawn in Fig. 1A to depict this laboratory detection level. Boxplot depicts median ± 1 IQR, with whiskers drawn to an additional 1.5 IQR from box limits (or to observed maximum/minimum values if these are closer). * signifies statistical difference between AIS and AIS + D groups at *p* < 0.05

Study laboratory test values are provided in a combined dot and box plot in Fig. 1a-d.

The detection threshold for IL-6 was 5 pg/mL; undetectable levels were assigned a numerical value of 0. Had undetectable levels been assigned a numerical value of 4 pg/mL, the median and IQR values for the AIS group would become 4 (1.5) while the median and IQR values for the AIS + D group would remain 11.5 (8.3). Statistical difference between groups would remain significant with *p* remaining 0.002.

## Discussion

Patients with underlying neurodegenerative dementia and acute ischemic stroke (AIS + D) had higher levels of serum IL-6 and CRP than patients with acute ischemic stroke alone (AIS). There was a trend towards higher serum S100B levels as well in the AIS + D group compared to the AIS group, but this difference did not reach statistical significance. No difference was seen in serum CC3 between the groups. There was one notably high value of IL-6 observed (107 pg/mL, with all other observed values ≤30). This was in a control (AIS) patient with a small right thalamic lacunar stroke. No obvious explanation for this elevated value was found upon chart review, other than possibly some contribution from a concurrent gastritis. Undetectable IL-6 levels (< 5 pg/mL) were assigned a numerical value of 0 for calculation purposes but this convenience did not affect the statistical probability of no difference (*p*-value) between the AIS and AIS + D groups (see Table 1 note, and depiction of detection threshold in Fig. 1a).

The AIS + D group differed significantly from the AIS controls in being older (85.8 ± 9.6 vs 64.5 ± 14.1 years old), having more severe stroke signs (initial NIHSS 11.9 ± 8.1 vs 4.3 ± 4.3), and having more atrial arrhythmias and congestive heart failure. In addition, one AIS + D patient but no AIS controls had a concurrent diagnosis of Parkinson’s disease. Any of these differences might have contributed to the elevated serum IL-6 and CRP levels found in the AIS + D group. However, age or atrial arrhythmias are unlikely to be main factors explaining the observed difference in these values. In a recent study examining older adults without acute illness (mean age 73, range 60–92), slight elevation in serum IL-6 was associated with a clinical judgement of “frailty” but all IL-6 measurements remained below 5 pg/mL [18]. A sampling of patients with ischemic stroke or transient ischemic attack showed a non-significant trend towards elevation in IL-6 in patients with atrial fibrillation compared to levels in patients with large artery atherosclerosis, but CRP was similar between groups (6.2 ± 5.4 mg/L vs 5.1 ± 4.2 mg/L, respectively), and lower than many of the CRP values seen here in AIS + D patients [19].

More severe strokes in the AIS + D group compared to the AIS group have greater potential to contribute to the observed elevations of serum IL-6 and CRP in the former group, as both IL-6 [20] and CRP [21] in blood are elevated by ischemic stroke. However, three considerations argue that elevations in these inflammatory biomarkers were not fully explained by infarct volume imbalance. First, the IL-6 values observed in the AIS + D group here (median 11.5 pg/mL with IQR 8.3) were higher than those observed by Hotter et al. in a series of 94 patients with acute stroke alone (median 2.8 pg/mL, IQR 3.2, on admission) [20], and similarly, the CRP values observed in the AIS + D here (median 19.8 mg/L with IQR 28.6) were higher than the values associated with severe strokes in the 316 patients studied by Luo et al. (≥ 7 mg/L) [21].

Second, serum levels of CC3 were not elevated in the AIS + D group compared to the AIS group. Blood levels of CC3 are elevated acutely after ischemic stroke due to either cardioembolic disease or small vessel occlusion [22–24]. Furthermore, serum levels of the astrocyte calcium-binding protein, S100B, also did not differ statistically between the AIS + D and AIS groups, although a trend towards higher levels in the AIS + D group was seen at *p* = 0.065. Serum S100B is also elevated after acute ischemic stroke, showing a positive correlation to infarct size [25, 26].

Lastly, an exploratory analysis in which only patients with NIHSS ≥10 was included in the AIS controls still showed significantly lower values of IL-6 (median 0, IQR 0) and CRP (median 4.3 mg/L, IQR 1.4) compared to values in the AIS + D group (*p* = 0.012 and 0.033, respectively) (Table 2).

**Table 2:** Exploratory analysis comparing the AIS + D group to a subgroup of AIS patients with similarly severe strokes

	AIS + D (*n* = 8)	mild AIS (*n* = 29)	*p*	severe AIS (*n* = 6)	*p*
IL-6 (pg/mL)	11.5 (9.8–18.0)	0 (0–6.0)	0.009	0 (0–0)	0.012
CRP (mg/L)	19.8 (13.2–41.8)	3.0 (1.0–10.0)	0.004	4.3 (3.6–5.0)	0.033

Note: Laboratory values are presented as median and range between 25th percentile and 75th percentile values, with between group comparisons between AIS + D and mild AIS or severe AIS controls performed using the Mann-Whitney *U* test. The mild AIS control group was comprised of AIS patients with NIHSS < 10; the severe AIS control group was comprised of AIS patients with NIHSS ≥10. Mean NIHSS in the severe AIS group was 12.3, similar to the mean NIHSS in the AIS + D group (11.9, Table 1)

Present data are thus consistent with the original hypothesis that opening of the BBB by acute ischemic stroke may permit the elevated levels of IL-6 and CRP present in the brain tissue of dementia patients to be reflected in the serum. Further study involving larger numbers of patients is warranted to test this hypothesis further.

In the present study, no attempt was made to refine the diagnosis of neurodegenerative dementia beyond clinical diagnosis. Based on population prevalence, a majority of the AIS + D group likely had Alzheimer’s disease, but other dementing illnesses may have been present in the group and one member of the AIS + D group also carried a diagnosis of Parkinson’s disease. As noted above, progressive neurodegenerative dementias in general are likely be associated with chronic brain inflammation. If opening of the BBB reliably enhances the appearance of brain tissue chemistry in serum, diagnostic BBB opening achieved with FUS might eventually be combined with serum measurements of a broader range of brain tissue biomarkers, including disease-specific biomarkers for AD and other neurodegenerative dementias.

## Conclusion

Patients with underlying neurodegenerative dementia and acute ischemic stroke had higher levels of serum IL-6 and CRP than patients with acute ischemic stroke alone. This observation is consistent with the possibility that opening of the BBB by ischemic stroke enhanced the appearance in blood of brain tissue markers of inflammation associated with neurodegenerative dementia. Further study is warranted to test this possibility, given the recent emergence of methods for transiently and safely opening the human BBB.

## Data Availability

Study data are available from the corresponding author upon request.

## Abbreviations

AD: Alzheimer’s disease
BBB: blood-brain barrier
FUS: low intensity focused ultrasound
IL-6: interleukin-6
CRP: C-reactive protein
S100B: S100B protein
CC3: Complement C3
